# Antimicrobial Prosthetic Surfaces in the Oral Cavity—A Perspective on Creative Approaches

**DOI:** 10.3390/microorganisms8081247

**Published:** 2020-08-17

**Authors:** Jorge L. Garaicoa, Amber M. Bates, Gustavo Avila-Ortiz, Kim A. Brogden

**Affiliations:** 1 Department of Restorative Dentistry, Oregon Health and Science University, School of Dentistry, Portland, OR 97201, USA and Escuela de Odontología, Universidad de Especialidades Espiritu Santo, Guayaquil 092301, Ecuador; garaicoa@ohsu.edu; 2 Department of Human Oncology, University of Wisconsin School of Medicine and Public Health, University of Wisconsin-Madison, Madison, WI 53705, USA; ambates@wisc.edu; 3 Department of Periodontics, College of Dentistry, The University of Iowa, Iowa City, IA 52242, USA; gustavo-avila@uiowa.edu; 4 Iowa Institute for Oral Health Research, College of Dentistry, The University of Iowa, Iowa City, IA 52242, USA

**Keywords:** antimicrobial, oral cavity, *Candida*, implants, polymicrobial biofilms, disinfectants, antibiotics, antimicrobial peptides

## Abstract

Replacement of missing teeth is an essential component of comprehensive dental care for patients suffering of edentulism. A popular option is implant-supported restorations. However, implant surfaces can become colonized with polymicrobial biofilms containing *Candida* species that may compromise peri-implant health. To prevent this, implant components may be treated with a variety of coatings to create surfaces that either repel the attachment of viable microorganisms or kill microorganisms on contact. These coatings may consist of nanoparticles of pure elements (more commonly silver, copper, and zinc), sanitizing agents and disinfectants (quaternary ammonium ions and chlorhexidine), antibiotics (cefalotin, vancomycin, and gentamicin), or antimicrobial peptides (AMPs). AMPs in bioactive coatings have a number of advantages. They elicit a protective action against pathogens, inhibit the formation of biofilms, are less toxic to host tissues, and do not prompt inflammatory responses. Furthermore, many of these coatings may involve unique delivery systems to direct their antimicrobial capacity against pathogens, but not commensals. Coatings may also contain multiple antimicrobial substances to widen antimicrobial activity across multiple microbial species. Here, we compiled relevant information about a variety of creative approaches used to generate antimicrobial prosthetic surfaces in the oral cavity with the purpose of facilitating implant integration and peri-implant tissue health.

## 1. Introduction

Replacement of missing teeth is an essential component of comprehensive dental care. The use of implant-supported restorations for tooth replacement has become increasingly popular for patients suffering of edentulism [1]. Unlike conventional tooth-supported fixed dental prostheses (FDPs), implant-supported restorations do not require the preparation of natural teeth at the expense of irreversibly eliminating healthy dental structure. Additionally, alveolar bone maintenance is promoted through the functional intraosseous stimulation that implants exert on the supporting tissues. Furthermore, implants have provided therapeutic versatility, since they can be used as fixtures for removable or fixed prostheses. Finally, long-term implant survival rates are high and have ranged from 85 to 98%, depending on the type of prosthetic restoration [2,3].

However, biomechanical and biological complications can arise leading to peri-implant disease [4]. Biomechanical failures have been associated with malfunction or deterioration of the implant body, fixation screw, abutment, or prosthetic crown, generally due to functional stress associated with parafunctional habits or trauma. Biological failures have been associated with the presence of polymicrobial biofilms that induce a local host inflammatory response in the surrounding tissues (peri-implant mucositis) [5]. If these complications are not resolved and inflammation is sustained over time, the peri-implant mucosal seal is eventually disrupted, facilitating the apical invasion of peri-implant tissues by microorganisms. Subsequently, the host inflammatory response may extend into the implant-supporting bone, leading to irreversible, progressive marginal bone loss and a clinical entity known as peri-implantitis [6]. Long-term clinical studies have reported an incidence of peri-implantitis ranging from 0.3% to 40.0% [7,8,9,10]. The reason for this wide range is likely due to the disparity in assessment methods used in different studies and the absence of a universal diagnostic criterion to identify the presence of peri-implantitis [11]. Risk and contributing factors for peri-implantitis have included prior history of poor oral hygiene, periodontitis, smoking, uncontrolled diabetes, and iatrogenic factors like residual cement [6,12].

In one study, the peri-implant and periodontal microbiomes from 81 individuals were found to be different; microorganisms that caused peri-implant disease were not necessarily the same as those that caused periodontal disease [13]. In fact, the majority of the abundant species were reported to be distinct between the implant and subgingival microenvironments. For example, *Staphylococcus* species and *Treponema* species were significantly associated with peri-implant disease, but not periodontal sites [13]. Furthermore, *Staphylococcus* species were elevated in certain individuals, but not consistently associated with peri-implant infections.

Although the peri-implant microbiome is unique, these biofilms establish on the surface of implanted materials in a similar manner to that on the natural dentition. A dental pellicle proteome is formed directly on the implant surface [14,15]. Early colonizing microorganisms then attach to the pellicle via van der Waals forces, electrostatic charges, and specific adhesive proteins [16]. If not removed, the cells proliferate and form colonies that produce an extensive extracellular matrix [17]. Late colonizers then attach and produce lipopolysaccharides (LPS), capsular polysaccharides, hemagglutinins, proteases, and toxins that are capable of inducing local tissue damage and induce inflammation characterized by the production of chemokines, proinflammatory cytokines, and matrix metalloproteinases [5,18,19].

Once established, biofilms are challenging to eradicate from implant surfaces. Therefore, preventing or slowing their development process would represent a plausible therapeutic strategy. These approaches may include altering the implant or abutment surface to prevent the initial attachment of host proteins and/or repel the attachment of early colonizers; coating implant materials with antimicrobial elements to kill microorganisms on contact; and coating implant materials with a matrix containing antimicrobial elements that are gradually released to kill microorganisms [7].

Here, we compiled relevant information about a variety of creative approaches used to generate antimicrobial prosthetic surfaces in the oral cavity with the primary purpose of facilitating implant integration and peri-implant tissue health. Some examples are hydrophilic, photocatalytic, and/or ion implanted surfaces to prevent the adhesion of bacterial cells, or nanostructured coated surfaces that slowly release nanoparticles of pure elements (e.g., silver, copper, or zinc); sanitizing agents and disinfectants including quaternary ammonium ions and chlorhexidine; the antibiotics cefalotin, vancomycin, and gentamicin; or antimicrobial peptides (AMPs) [20,21]. A comprehensive synopsis of these antimicrobial agents in implant coatings is listed in Table 1, Table 2, Table 3 and Table 4.

## 2. Elements in Bioactive Coatings

Pure elements and metal salts have been used as effective antimicrobial agents against pathogenic microorganisms, including oral microorganisms and periodontal pathogens [20,22]. Antibacterial properties and their effectiveness to prevent bacterial attachment may differ significantly among different elements, depending on the target bacterial species [23]. Titanium and tin generally do not exhibit much antibacterial activity, whereas cobalt, nickel, copper, zinc, zirconium, and molybdenum do and, therefore, show promise as potential surface coatings of titanium implants (Table 1).

Silver appears to be the most promising element, with strong antimicrobial activity while exhibiting minimal cytotoxicity to human oral cells [20]. For example, nanoparticles or composite nanoparticles of silver coated on titanium or incorporated into polyacrylate-based hydrogel or lactose-modified chitosan coatings on titanium inhibited Gram-positive and Gram-negative bacteria with little cytotoxicity to osteoblast-like cells and primary human fibroblasts [24,25,26,27,28]. Silver was also effective when incorporated into resins and dental materials. Silver nanoparticles (10–200 ppm) in denture liner material and silver nanoparticles (20.0–30.0%) in denture acrylic disks inhibited the growth of *Candida albicans* [29,30]. Salts of silver have also exhibited antimicrobial capacity and 0.5% silver benzoate in resins and 0.5–25.0 µg/mL silver nitrate applied topically inhibited *Streptococcus mutans, Porphyromonas gingivalis*, *Prevotella intermedia*, *Treponema denticola*, *Tannerella forsythia*, *Fusobacterium nucleatum ss vincentii, Campylobacter gracilis*, *Campylobacter rectus*, *Eikenella corrodens***,** and *Aggregatibacter actinomycetemcomitans* [22,31]. Differing methods have been used to prepare these nanoparticles. For example, Shankar et al. used Neem (*Azadirachta indica*) leaf broth to enhance the synthesis of gold nanoparticles, silver nanoparticles, and bimetallic gold/silver nanoparticles [38]. Ultrastructurally, a core–shell structure was formed; silver nanoparticles were on gold nanoparticles. They thought that this synthesis method could achieve rates of synthesis equal to that of conventional chemical methods.

Different studies have shown that other elements display antimicrobial activity with minimal cytotoxicity. Copper oxide nanocomposites in polyurethane inhibited methicillin-resistant *Staphylococcus aureus* (MRSA) [33]; hydrogenated copper films inhibited *S. aureus* and *Escherichia coli,* but not the adhesion and proliferation of MG-3 osteoblasts and NIH-3Te fibroblasts [34]; and zinc oxide in coatings inhibited *S. aureus* and *E. coli,* but were compatible with the adhesion, proliferation, and differentiation of rat bone marrow stem cells [35]. Bismuthinetriyltris(oxy) tris(oxoazane) trioxide (BiN_3_O_9_) and bismuth (3+) triacetate (C_6_H_9_BiO_6_) on titanium disks inhibited *A. actinomycetemcomitans* and MRSA but were not cytotoxic for MG63 osteoblast-like cells [36]. Selenium carbonated hydroxyapatite coatings prevented the establishment of *P. aeruginosa* and *S. aureus* biofilms, were not cytotoxic to MC3T3-E1 preosteoblasts, and enhanced cell proliferation [37]. Cobalt, copper, zinc, zirconium, molybdenum, and lead in resin resulted in a fourfold reduction of *S. aureus* and *E. coli* within 24 h compared to controls [23].

Diogo et al. assessed antimicrobial activity and cytotoxicity of a chlorophyll-based photosensitizer Zn(II)chlorine methyl ester ((Zn(II)e_6_Me) [39]. The chlorophyll derivative Zn(II)e_6_Me was antimicrobial for mixed biofilms and had minimal cytotoxicity for human apical papilla cells.

## 3. Antiseptics and Disinfectants in Bioactive Coatings

Antiseptics and disinfectants are effective antimicrobial agents [40] when incorporated in polymers and implant coating materials (Table 2). They rapidly kill microorganisms via disruption of phospholipid bilayers; removal of divalent cations; release of LPS; disruption of cross-linked proteins in the cell membrane; inactivation of membrane-bound enzymes; and damage to cross-linked intracellular proteins, RNA, and DNA [40].

Compounds containing chlorhexidine, a strong disinfectant that prevents initial bacterial adhesion, have been applied in calcium phosphate phases to titanium alloy (e.g., Ti6Al4V) surfaces [41]. These surfaces were formed by a co-deposition process of both phases where chlorhexidine interacted with the deposition and transformation of calcium phosphate phases in the coating. For high chlorhexidine contents, coatings consisted of chlorhexidine crystals coated by nanocrystalline hydroxyapatite [41].

Disinfectants of the guanidine and polyhexanide families are also very effective against a variety of Gram-positive and Gram-negative bacteria and have potential as effective antimicrobial agents against oral microorganisms [42]. Guanidine incorporated into homo- and copolymers of 2-aminoethylmethacrylate in solution or in coatings were not cytotoxic to cells and inhibited *E. coli* and *Bacillus subtilis* [42]. Half maximal inhibitory concentration (IC50) was 9 µg/mL and minimal inhibitory concentrations (MIC) were 16 µg/mL. Polyhexanide encapsulated in hyaluronic acid inhibited hyaluronidase producing stains of *S. aureus* (MIC, 62.5 µg/mL) and *E. coli* (MIC, 250.0 µg/mL) [43].

Although not cytotoxic for human gingival fibroblasts, compounds containing quaternary ammonium, phosphonium, or pyridinium ions, like poly-(4-vinyl-N-hexylpyridinium bromide) appear to be weaker disinfectants and implant coatings have only moderate antimicrobial activity for oral microorganisms like *Streptococcus mutans* or *Streptococcus sanguinis* [44].

## 4. Antibiotics in Bioactive Coatings

Antibiotics can also be used in bioactive coatings to prevent peri-implant diseases (Table 3). Antibiotics may act by altering microbial cell wall, nucleic acid, or protein synthesis, resulting in cell envelope damage and loss of structural integrity; double stranded DNA breaks; inactivation of DNA-dependent RNA synthesis; disrupted cellular energetics; and/or production of harmful hydroxyl radicals involving alterations in central metabolism [48]. Antibiotics that act on microbial membranes and/or inhibit cell wall or protein synthesis have been used as effective constituents in bioactive coatings to prevent peri-implant diseases (Table 3). Generally, two strategies have been adopted. One strategy involved the continued slow release of antibiotics from coatings containing free antibiotics. Vancomycin incorporated into chitosan and deposited onto titanium to be released in a biologically active form inhibited the growth of *S. aureus* [49]; vancomycin incorporated into polymer multilayer films inhibited the growth of *S. aureus* [50]; and cefalotin incorporated into apatite and deposited onto titanium inhibited *S. mutans* [51].

Another strategy involved the immobilization of antibiotics to implant surfaces. This strategy worked well to repel the attachment of viable microorganisms or kill microorganisms on contact with the implant surface to prevent the onset of peri-implant mucositis. Protein synthesis inhibitors like tobramycin, gentamicin, and tetracycline have been used for this purpose. Tobramycin immobilized on microporous octacalcium phosphate on titanium inhibited *P. aeruginosa* over 4 h [52]. Gentamicin incorporated into poly d,l-lactide polymeric coatings inhibited the growth of *S. aureus* [53], while 20.0% tetracycline incorporated into chitosan coatings and bonded to titanium (737.0 + 125.7 µg/cm^2^) inhibited *S. epidermidis* and *A. actinomycetemcomitans,* but was not cytotoxic to osteoblastic cells or fibroblasts [45].

Nystatin and Amphotericin B are antibiotics that act on fungal membranes. They bind to ergosterol and form concentration-dependent transmembrane pores. Potassium, sodium, hydrogen, and chloride ions leak from the cytoplasm leading to eventual fungal cell death. Both 1.0% nystatin and 0.1% Amphotericin B in polymers applied to denture materials inhibited *C. albicans* biofilms [46].

Rai et al. synthesized spherical gold nanoparticle (52–22 nm) and cefaclor complexes [32]. This complex had potent antimicrobial activity against both *S. aureus* and *E. coli* with MICs of 10 mg/mL and 100 mg/mL, respectively. The combined action of Au and cefaclor were thought to interfere with the synthesis of peptidoglycan and creating pores in the microbial cell walls.

## 5. AMPs in Bioactive Coatings

AMPs are relatively small peptides that contain cationic, anionic, or amphipathic amino acid residues. These peptides attach to microbial surfaces and may kill microorganisms by a variety of mechanisms: formation of lethal pores; disruption of membrane integrity; or cytoplasmic penetration to inhibit cell wall synthesis, alteration of septum formation in the cytoplasmic membrane, binding to DNA, inhibition of enzymatic activity, DNA, RNA, and/or protein synthesis [54]. AMPs are widely distributed throughout species of the Monera (e.g., Eubacteria), Protista (e.g., protozoans and algae), Fungi (yeasts), Plantae (plants), and Animalia (e.g., insects, fish, amphibians, reptiles, birds, and mammals) kingdoms [55]. They include groups of synthetic peptide mimetics, hybrid peptides, peptide congeners, stabilized peptides, peptide conjugates, and immobilized peptides [56]. Natural, as well as synthetic AMPs derived from lactoferrin [52,57], LL-37 [58], and beta defensins [59], may be active against *Candida* species and other microorganisms, and have the potential to be applied in bioactive implant coatings (Table 4).

AMPs can be adsorbed to surfaces, coated on surfaces, or covalently bonded to functionalized surfaces to prevent microbial biofilm formation. These alternatives open the door to different therapeutic strategies. One strategy is to incorporate AMPs into films for continuous release over time at relevant concentrations. Films that allow heavy drug loading and favorable release kinetics to prevent attachment of microorganisms are desirable [50]. Another strategy is to use nondiffusible AMPs by means of covalent immobilization to material surfaces. It has been shown that AMPs tethered to functionalized poly-(ethylene glycol) can form a nonadhesive peptide layer that kills bacteria on contact [58]. AMPs immobilized to titanium surfaces have been tested to shorten the period of osseointegration and to reduce colonization of periodontopathogens to implant surfaces [60,61,62]. Immobilized histatin alone or conjugated peptides of histatin 5/titanium binding peptide and lactoferricin/titanium binding peptide reduced colonization of *P. gingivalis* and enhanced mRNA expression of runt-related transcription factor 2 (Runx), Osteopontin (OPN), and alkaline phosphatase (ALPL) in osteoblastic cells. Runx2 is an inducer of osteoblast and chondrocyte differentiation [63], OPN is important in cell communication and matrix mineralization [64], and ALPL is involved cementum mineralization [65]. AMP Tet213 loaded on a thin microporous coating of calcium phosphate on titanium had antimicrobial activity against Gram-positive and Gram-negative bacteria with 10^6^-fold reductions in CFU within 30 s [52,66]. It has been also demonstrated that calcium phosphate–Tet213 is a more efficient antimicrobial coating than calcium phosphate–MX226, calcium phosphate–hLF1-11, or calcium phosphate–tobramycin.

Following incubation of calcium phosphate-coated implants with equimolar concentrations of Tet213, the commercially developed antimicrobial peptide MX-226, hLF1-11, or tobramycin inhibited bacterial growth. Also, Chrysophsin-1 and -3 incorporated into acrylic coating systems exhibited antimicrobial activity against both Gram-positive and Gram-negative bacteria in vitro, which may be applicable for future dental applications to reduce bacterial colonization and a subsequently favorable tissue response [67].

The mechanisms of activity of AMPs bound to surfaces or incorporated into materials remain unknown [54]. The chemical coupling procedure, length of spacers, peptide orientation, and peptide concentration are all important contributing factors [69]. The antimicrobial activity distinctly decreased with reduction of the spacer length. Still, peptides are thought to insert into the target membrane by using an exchange of membrane-stabilizing bivalent cations, which contributed to the antimicrobial effect [70]. Other studies suggest that the surface actions of AMPs were sufficient for their lethal activities [71].

## 6. Antimicrobial Substances with Future Potential in Bioactive Coatings

There are other antimicrobial substances that have potential as additives in bioactive coatings to prevent peri-implant diseases due to their proven activity against oral microorganisms. Phenylalkyne compounds, arylamide compounds, and the mimetic mPE have potent antifungal activity against planktonic cultures and biofilms of *Candida* species [72] and antimicrobial activity against biofilms of *A. actinomycetemcomitans* and *P. gingivalis* [73]. It has also been shown that *S. mutans* competence stimulating peptide (CSP) attached to an antimicrobial peptide domain can kill *S. mutans* [74]. We found that coupling an antibody specific to the outer surface of *P. gingivalis* strain 381 to sheep myeloid antimicrobial peptide (SMAP28) selectively killed *P. gingivalis* in an artificially generated microbial community containing *P. gingivalis*, *A. actinomycetemcomitans*, and *P. micros* [75]. In other studies, AMPs have also been tethered to resins or brush layers with proposed uses as contact-active cationic antimicrobial surfaces [70,71].

Furthermore, AMPs and lysozyme have been encapsulated within silica or titanium nanoparticles to create bio-nano-composite materials with antimicrobial activity for use as broad-spectrum antifouling materials or in cosmetics with sunscreen properties [76]. Another AMP suitable for incorporation into coatings for dental implants is human lactoferrin 1-11 (hLF1-11), which is derived from the first 11 amino acids of human lactoferrin [57].

Some antimicrobials have shown promise in combination with bone regeneration enhancers. Surfaces with co-immobilized arginylglycylaspartic acid (RGD) and PHSRN peptides were found to significantly improve osteoblast responses [77]. Surfaces containing gentamicin and bone morphogenetic protein-2 showed enhanced antibacterial activity and osseointegration compared to a control [78].

Some lipids are also active against oral microorganisms and have potential as additives to bioactive coatings or creams to prevent peri-implant and other oral diseases. Long chain bases (sphingosine, dihydrosphingosine, and phytosphingosine) and short chain fatty acids (sapienic acid and lauric acid) have exhibited antimicrobial activity against a variety of Gram-positive bacteria, Gram-negative bacteria, and oral bacteria, by inducing ultrastructural damage and altered microbial metabolism [79,80,81]. Recent work suggests these lipids are also likely involved in innate immune defense against epidermal and mucosal bacterial infections [82,83]. However, little is known yet about the spectrum of lipid activity against oral bacteria and *Candida* species or their mechanisms of action.

## 7. Future Directions

Development and testing of prosthetic surface coating strategies represents a relevant and emerging topic in contemporary research with great potential for therapeutic application in dentistry and other clinical disciplines. Although promising results have been reported, the vast majority of the evidence available to date emanates from in vitro studies and each approach has advantages and disadvantages.

For endodontic applications, a promising methodology is the use of photodynamic therapy (PDT) as an alternative to classical endodontic irrigation solutions and antibiotics for the treatment of apical periodontitis [84,85]. Diogo et al. first assessed the ability of PDT to improve root canal disinfection [84]. They concluded that the antimicrobial activities of PDT were most effective when used with toluidine blue and methylene blue at 660 nm wavelength with a 400 nm diameter of intracanal fiber. Diogo et al. then assessed the antimicrobial approaches to improve PDT efficiency [85]. Two favorable approaches emerged for endodontic purposes that included drug delivery systems using nanoparticles and photosensitizer solubilizers.

For oral implantology applications, antimicrobial prosthetic surfaces should not only prevent bacterial adhesion, but allow or even enhance the attachment, proliferation, and differentiation of host cells to promote adequate peri-implant healing and long-term health [86]. Future investigations in this field should focus on clinical translation with the purpose of assessing the performance and biosafety of different antimicrobial coatings aimed at reducing microbial growth and biofilm formation on implant prosthetic surfaces.

## Figures and Tables

**Table 1:** Elements with antimicrobial activities used in bioactive coatings.

Element	Modified Material	Antimicrobial Activity	Pathogen(s)	References
Silver (Ag)	Ag nanoparticle–TiO_2_ composite	Growth inhibition	Methicillin-resistant *Staphylococcus aureus* (MRSA)	[24,25,26]
	Ag nanoparticle–polyacrylate hydrogel	Growth inhibition	Gram-positive, Gram-negative bacteria	[27]
	Ag nanoparticles, chitosan	Growth inhibition	*Staphylococcus aureus*, *Pseudomonas aeruginosa*	[28]
	Ag nanoparticles	16.3–52.5% inhibition	*Candida* *albicans*	[29]
	Ag nanoparticles (20.0–30.0%)	Growth inhibition	*C. albicans*	[30]
	Ag benzoate (0.2 and 0.5%)	52.4–97.5% inhibition	*Streptococcus mutans*	[31]
	Ag nitrate (0.5 and 25.0 µg/mL)	Growth inhibition	*S. mutans, Porphyromonas gingivalis*, *Prevotella intermedia*, *Treponema denticola*, *Tannerella forsythia*, *Fusobacterium nucleatum ss vincentii, Campylobacter gracilis*, *Campylobacter rectus*, *Eikenella corrodens*, *Aggregatibacter actinomycetemcomitans*	[22]
Gold (Au)	Au nanoparticles, cefaclor	Growth inhibition	*S. aureus*, *Escherichia coli*	[32]
Cobalt (Co)	Co in resin	Growth inhibition	*S. aureus*, *E. coli*	[23]
Copper (Cu)	Cu oxide nanocomposites, polyurethane	Growth inhibition	MRSA	[33]
	Cu in diamond-like carbon film	Growth inhibition	*S. aureus*, *E. coli*	[34]
	Cu in resin	Growth inhibition	*S. aureus*, *E. coli*	[23]
Zinc (Zn)	ZnO coating	Growth inhibition	*S. aureus*, *E. coli*	[35]
	Zn in resin	Growth inhibition	*S. aureus*, *E. coli*	[23]
Zirconium (Zr)	Zr in resin	Growth inhibition	*S. aureus*, *E. coli*	[23]
Bismuth (Bi)	BiN_3_O_9_, C_6_H_9_BiO_6_ coating	Growth inhibition	*A. actinomycetemcomitans*, MRSA	[36]
Selenium (Se)	Se in hydroxyapatite coating	Growth inhibition	*P. aeruginosa*, *S. aureus* biofilms	[37]
Molybdenum (Mo)	Mo in resin	Growth inhibition	*S. aureus*, *E. coli*	[23]
Lead (Pb)	Pb in resin	Growth inhibition	*S. aureus*, *E. coli*	[23]

**Table 2:** Antiseptics and disinfectants used in bioactive coatings.

Antiseptic, Disinfectant	Material	Antimicrobial Activity	Pathogen	References
Poly-(4-vinyl-N-hexyl pyridiniumbromide)	Coating	No inhibition	*Streptococcus mutans* *, * *Streptococcus* *sanguinis*	[44]
Chlorhexidine (CHX)	0.02% CHX digluconate, chitosan coating	95–100% inhibition	*Staphylococcus epidermidis*	[45]
	0.02% CHX digluconate, chitosan coating	0–56% inhibition	*Aggregatibacter* *actinomycetemcomitans*	[45]
	1.0% CHX digluconate polymer-based coating	98% inhibition	*Candida albicans* biofilm	[46]
Fluoridated hydroxyapatite	Fluoridated calcium phosphate coating	51.6–82.3% inhibition	*Staphylococcus aureus, Escherichia coli, * *Porphyromonas* *gingivalis*	[47]
Polyhexanide	Hyaluronic acid, polyhexanide nanocapsules	62.5–250.0 µg/mL MIC	*S. aureus*, *E. coli*	[43]
Guanidine	2-aminoethyl-methacrylate polymer	16 µg/mL MIC	*E. coli*, *Bacillus subtilis*	[42]

MIC: minimum inhibitory concentration.

**Table 3:** Antibiotics used in bioactive coatings and their antimicrobial activities.

Antibiotic	Material	Antimicrobial Activity	Pathogen	References
Cefaclor (CEC)	Gold (Au) nanoparticles, cefaclor	Growth inhibition	*Staphylococcus aureus, Escherichia coli*	[32]
Cefalotin (CET)	CET, apatite	Growth inhibition	*Streptococcus mutans*	[51]
Tobramycin (TOB)	TOB, octacalcium phosphate layer	Growth inhibition	*Pseudomonas aeruginosa*	[52]
Vancomycin (VAN)	VAN, chitosan	Growth inhibition	*S. aureus*	[49]
	VAN, polymer films	Growth inhibition	*S. aureus*	[50]
Gentamicin (GEN)	GEN, poly(d,l-lactide) coating	Growth inhibition	*S. aureus*	[53]
Tetracycline (TET)	20.0% TET, chitosan coating	94–99% inhibition	*Staphylococcus epidermidis, * *Aggregatibacter* *actinomycetemcomitans*	[45]
Nystatin (NYT)	1.0% NYT, polymer coating	74–75% inhibition	*Candida albicans* biofilm	[46]
Amphotericin B (AMB)	0.1% AMB, polymer coating	49–55% inhibition	*C. albicans* biofilm	[46]

**Table 4:** Antimicrobial peptides used in bioactive coatings and their antimicrobial activities.

Antimicrobial Peptide	Material	Antimicrobial Activity	Pathogen	References
Tet213	Tet213, octacalcium phosphate layer	Growth inhibition	*Pseudomonas aeruginosa*	[52]
Mx226	Mx226, octacalcium phosphate layer	Growth inhibition	*P. aeruginosa*	[52]
human lactoferrin 1-11 (hLF1-11)	hLF1-11, octacalcium phosphate layer	Growth inhibition	*P. aeruginosa*	[52]
HHC36	HHC36, octacalcium phosphate layer	Growth inhibition	*P. aeruginosa* and *Staphylococcus aureus*	[52]
Carboxy-terminal leucine/isoleucine heptad repeat-1,3 (Chr-1,3)	Chr-1, 3 in acrylate 50% w/w resin	Growth inhibition	*S. aureus, Escherichia coli*	[67]
Human β defensin 2 (HBD2)	HBD2, methoxy silane	60–100% inhibition	*E. coli*	[59]
Ponericin (G1)	Ponericin G1 in multilayer films	Growth inhibition	*S. aureus*	[68]
Cathelicidin (LL-37)	LL-37 surface peptide layer	Growth inhibition	Bacteria	[58]
